# Adverse Reactions after BNT162b2 Messenger RNA Vaccination for Coronavirus Disease 2019 in Healthcare Workers Compared with Influenza Vaccination

**DOI:** 10.3390/vaccines11020363

**Published:** 2023-02-05

**Authors:** A-Sol Kim, Sung-Min Kim, Ji-Eun Song, Soyoon Hwang, Eunkyung Nam, Ki Tae Kwon

**Affiliations:** 1 Department of Family Medicine, Kyungpook National University Chilgok Hospital, School of Medicine, Kyungpook National University, Daegu 41404, Republic of Korea; 2 Division of Infectious Diseases, Department of Internal Medicine, Kyungpook National University Chilgok Hospital, School of Medicine, Kyungpook National University, Daegu 41404, Republic of Korea

**Keywords:** SARS-CoV-2, health personnel, immunization, injection site reaction, fatigue

## Abstract

This study aimed to compare adverse reactions following BNT162b2 and influenza vaccinations in healthcare workers. This study included healthcare workers who received the BNT162b2 vaccine and/or inactivated influenza vaccine, quadrivalent (IIV4), on 18–29 October 2021 at a tertiary hospital in Korea. IIV4 was administered and BNT162b2 was subsequently administered one week later. The participants responded to a mobile questionnaire regarding adverse events. The overall adverse reaction rates were 90.6% in the BNT162b2 + IIV4 group, 90.4% in the BNT162b2 alone group, and 44.1% in the IIV4 alone group (*p* < 0.001). Fever occurred in 19.5%, 26.9%, and 3.3% of participants in the BNT162b2 + IIV4, BNT162b2 alone, and IIV4 alone groups, respectively (*p* < 0.001). The most common local and systemic adverse reactions were injection site pain (65.0%) and fatigue (58.6%), respectively. Injection-site pain was experienced by 88.7%, 88.5%, and 37.5% of the BNT162b2 + IIV4, BNT162b2 alone, and IIV4 alone groups, respectively (*p* < 0.001). Fatigue was experienced by 74.8%, 78.8%, and 38.6% of the BNT162b2 + IIV4, BNT162b2 alone, and IIV4 alone groups, respectively (*p* < 0.001). Adverse reactions occurred at a significantly higher frequency after BNT162b2 than after IIV4. The frequency of adverse reactions one week after vaccination with IIV4 and BNT162b2 was not different from that after vaccination with BNT162b2 alone. Therefore, coadministration of influenza vaccine with BNT162b2 can be expected to be safe.

## 1. Introduction

Coronavirus disease 2019 (COVID-19) is caused by the newly identified severe acute respiratory syndrome coronavirus 2 (SARS-CoV-2). This disease was first reported in December 2019 as pneumonia of unknown cause in Wuhan, China. Owing to its rapid spread and severe progression, the World Health Organization declared COVID-19 a pandemic in March 2020. To pull through this pandemic situation, considerable efforts have been exerted worldwide into the development of a COVID-19 vaccine, and vaccine development has occurred faster than ever before. The first COVID-19 vaccine was administered in the United Kingdom in December 2020.

Nonetheless, infection persists even in countries with high vaccination rates, such as Israel. Additionally, the frequency of breakthrough infection, which becomes an infection after the completion of vaccination, has increased, and the appearance of Delta variants seems to have rendered the vaccine less effective [1]. Accordingly, the need for a booster shot has emerged [2]. In Israel, booster vaccination was approved in July 2021. The concentration of neutralizing antibodies was confirmed to be 10 times higher after booster vaccination than after administration of the second dose [3].

Despite the rapidly increasing vaccination rate in Korea, community transmission persists, thereby increasing the need for booster vaccination. Therefore, from October 2021, booster vaccination with a nucleoside-modified messenger RNA (mRNA)-based vaccine was planned and implemented first in high-risk groups and hospital workers. Subsequently, booster vaccinations were sequentially provided to all citizens. Booster vaccination with mRNA vaccines exhibited beneficial effects against the Omicron (B1.1.529) variant of SARS-CoV-2, which is currently a global pandemic [4]. As of May 2022, approximately 33 million Koreans had completed the third dose of COVID-19 vaccination and a fourth dose of vaccination for high-risk groups was in progress [5].

Adverse local reactions commonly observed after COVID-19 vaccination include injection site pain, swelling, and redness. Fever, fatigue, headache, and myalgia may occur as adverse systemic reactions. These adverse reactions may occur during a normal immune response [6]. Adverse events such as myocarditis/pericarditis, anaphylaxis, and thrombosis with thrombocytopenia syndrome need to be carefully observed; however, their incidence has been less high in Korea [7].

A previous study investigating adverse reactions following BNT162b2 mRNA vaccination in healthcare workers in Korea reported that the frequency of adverse reactions was higher after the second dose than after the first dose [8]. A phase 3 clinical trial of the third dose in 5081 patients revealed that local and systemic reactogenicity events were of low grade and no new safety signals were identified compared with the first and second doses of vaccination [9]. Nevertheless, real-world data on the safety of the third dose are insufficient. When SARS-CoV-2 infection becomes endemic after being a global pandemic, substantial medical and social burdens may be imposed if local epidemics are repeated along with the outbreak of seasonal infectious diseases, such as influenza. Therefore, vaccination against these diseases is exceedingly important. In light of the above, the frequency and severity of adverse reactions following the simultaneous or sequential performance of influenza vaccination and the third dose of BNT162b2 mRNA vaccination at short intervals (within 14 days) need to be investigated.

Hence, the present study aimed to investigate adverse reactions that could occur when the third dose of BNT162b2 is administered to adults who have received the second dose of BNT162b2 before 6 months. Furthermore, this study compared and analyzed the frequency and severity of adverse reactions according to whether or not influenza vaccination was performed together with booster vaccination.

## 2. Materials and Methods

### 2.1. Study Design and Participants

**Figure 1:**
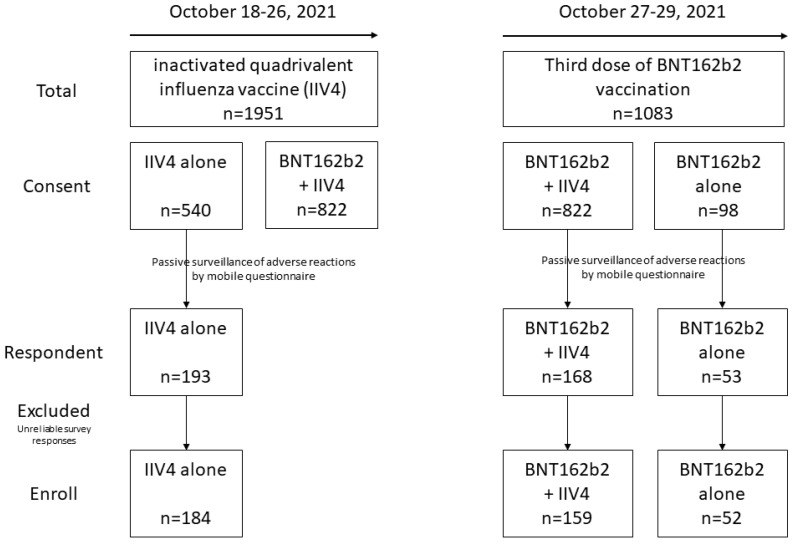
Study enrollment.

This study was conducted on healthcare workers (HCWs) who completed the BNT162b2 (Comirnaty™; Pfizer-BioNTech, New York, NY, USA) vaccination 6 months ago at a tertiary hospital in Daegu, Korea. Egg-based, split-virion, inactivated influenza quadrivalent vaccine (IIV4) (Boryung FLU Vaccine VIII-TFinj^®^; Boryung Biopharma, Jinchoen, Chungcheongbuk-do, Korea) was administered in these HCWs, followed by the third dose of the BNT162b2 vaccine 7 days later. Each vaccination was administered according to the manufacturer’s instructions within the period of 18–29 October 2021. Based on their preference, the HCWs selected either IIV4 alone, both IIV4 and BNT162b2 booster vaccination, or BNT162b2 booster vaccination alone. Those who developed serious allergic reactions to a previous BNT162b2 immunization and/or IIV4 or refused to receive vaccination for personal reasons were excluded. After 7 days from the date of each vaccination, the participants who consented to the study received a mobile questionnaire regarding adverse events that occurred within 7 post-vaccination days (Figure 1). Participants with unreliable responses (e.g., responding with symptoms and then responding with no symptoms in the sub-questions) were excluded through consensus among all authors.

### 2.2. Data Collection

The following data were investigated: participants’ sex and date of birth, type of vaccination, presence or absence of adverse reactions after a previous BNT162b2 vaccination, and antipyretic drug use and associated adverse reactions. The adverse reactions investigated via questionnaire included 13 systemic symptoms (fever, headache, chills, fatigue, muscle pain, arthralgia, dizziness, nausea and vomiting, chest pain, abdominal pain, diarrhea, anxiety, and worsening of mental symptoms [e.g., anxiety/depression]), 3 local symptoms (injection site pain, redness, and edema), and other symptoms that were freely described. With respect to severity, adverse reactions were graded as follows: mild (transient or mild discomfort, no interference with daily activities, and no requirement of medical intervention or therapy); moderate (mild-to-moderate limitations in daily activities and no or minimal requirement of medical intervention or therapy); severe (substantial limitations in daily activities and requirement of medical intervention or therapy); and potentially life-threatening (required assessment in the emergency department or admission to a hospital). The questionnaire sheet used in this study is provided as a Supplementary Materials.

### 2.3. Statistical Analyses

A descriptive analysis was conducted on the participants. The rate of adverse reactions according to the type of vaccination was compared using analysis of variance for continuous variables and Chi-square or Fisher’s exact tests for nominal variables. All statistical analyses were performed using R version 3.6.1 (The R Foundation for Statistical Computing, Vienna, Austria). Statistical significance in all analyses was set at *p* < 0.05.

### 2.4. Ethics Statement

The present study was conducted in accordance with the principles embodied in the Declaration of Helsinki. This study was reviewed and approved by the Daegu Joint Institutional Review Board (IRB approval no.: DGIRB 2021-10-001-001). Written informed consent was obtained from all individual participants included in this study.

## 3. Results

### 3.1. Demographics

Among 2061 HCWs (575 men and 1486 women) who consented to participate in the study, 822 received combined vaccinations (IIV4 + BNT162b2), 98 received the BNT162b2 booster vaccination alone, and 540 received IIV4 alone. Among those who consented to participate in this study, 159 (19.3%), 52 (53.1%), and 183 (33.9%) HCWs in the IIV4 + BNT162b2 group, BNT162b2 alone group, and IIV4 alone group, respectively, responded to the questionnaire. The mean age was 35.68 ± 8.69 years in the IIV4 + BNT162b2 group, 39.27 ± 9.21 years in the BNT162b2 alone group, and 34.12 ± 9.32years in the IIV4 alone group. Males accounted for 18.9% of the IIV4 + BNT162b2 group, 32.7% of the BNT162b2 alone group, and 22.3% of the IIV4 alone group (Table 1).

### 3.2. Adverse Reactions within 7 Days after Vaccination

The overall adverse reaction rates were 90.6%, 90.4%, and 44.1% in the IIV4 + BNT162b2 group, BNT162b2 alone group, and IIV4 alone group, respectively. The systemic adverse reaction rates were 78.0%, 80.8%, and 38.6% in the BNT162b2 + IIV4 group, BNT162b2 alone group, and IIV4 alone group, respectively (*p* < 0.001). No significant differences in the overall and systemic adverse reaction rates were noted between the BNT162b2 + IIV4 and BNT162b2 alone groups (*p* = 0.519). The most common local and systemic adverse reactions were injection site pain (65.0%) and fatigue (58.6%), respectively. In particular, 88.7%, 88.5%, and 37.5% of the BNT162b2 + IIV4, BNT162b2 alone group, BNT162b2 alone group, and IIV4 alone group, respectively, experienced injection site pain (*p* < 0.001). Additionally, 74.8%, 78.8%, and 38.6% of the BNT162b2 + IIV4 group, BNT162b2 alone group, and IIV4 alone group, respectively, developed fatigue (*p* < 0.001). Fever occurred in 19.5%, 26.9%, and 3.3% of participants in the BNT162b2 + IIV4 group, BNT162b2 alone group, and IIV4 alone group, respectively (*p* < 0.001). The three groups did not show any significant differences with respect to vomiting (*p* = 0.132), diarrhea (*p* = 0.276), and abdominal discomfort (0.059) as adverse reactions (Table 2).

### 3.3. Significant Adverse Reactions According to the Date after Vaccination

**Figure 2:**
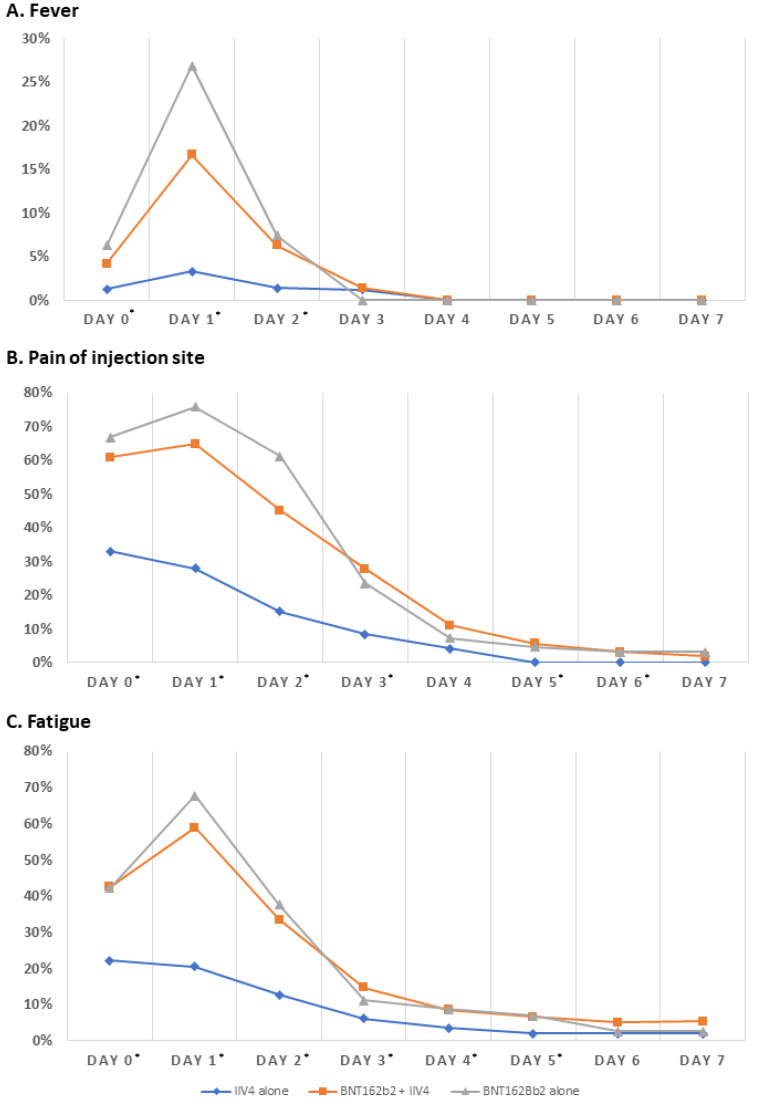
Frequency of significant adverse reactions according to the date after each vaccination. The frequency of significant adverse reactions within 7 days after each vaccination is shown. (**A**) Fever. (**B**) Injection site pain as the most common local reaction. (**C**) Fatigue, which was the most common systemic adverse reaction. The asterisk (*) after the date indicates a significant difference between the 3 groups. IIV4 = inactivated influenza vaccine, quadrivalent.

Fever on the day of vaccination occurred in 4.2% of the BNT162b2 + IIV4 group, 6.3% of the BNT162b2 alone group, and 1.3% of the IIV4 alone group, which reached statistical significance (*p* < 0.001). The incidence of fever was highest on the first day after vaccination; specifically, 16.7%, 26.9%, and 7.4% of the BNT162b2 + IIV4 group, BNT162b2 alone group, and IIV4 alone group, respectively, developed fever (*p* < 0.001). On the second day after vaccination, fever occurred in 16.7% to 26.9% of those who received BNT162b2, regardless of whether or not IIV4 was administered; however, the rate decreased thereafter. In the post-hoc analysis performed to analyze the difference in incidence between the groups, no statistical significance was observed between the BNT162b2 + IIV4 group and IIV4 alone group on the day of vaccination and on the second day after vaccination (*p* = 0.197 for day 0 and *p* = 0.790 for day 2). Both groups showed a statistically significant difference from the IIV4 alone group with respect to the incidence of fever (Figure 2A).

The incidence of injection site pain, which was the most common local reaction, was highest on the day of injection in the BNT162b2 alone group (66.8%), followed by the BNT162b2 + IIV4 group (60.9%) and IIV4 alone group (33.1%). There was no significant difference between the BNT162B2 + IIV4 group and the BNT162b2 alone group (*p* = 0.486), and these 2 groups exhibited a statistically significant difference from the IIV4 alone group (*p* < 0.001 for BNT162b2 + IIV4 group vs. IIV4 alone group and *p* < 0.001 for BNT162b2 alone group vs. IIV4 alone group). Similar to fever, the incidence of injection site pain was highest on the first day after injection; however, the incidence declined to <20% in all three groups after the fourth day after injection. In the IIV4 alone group, none of the participants complained of injection site pain after the fifth day after injection; in contrast, those who received BNT162b2 vaccination complained of injection site pain until the seventh day (Figure 2B).

The incidence of fatigue, which was the most common systemic reaction, was highest on the first day after injection. The BNT162b2 + IIV4 group and the BNT162b2 alone group did not statistically differ (*p* = 0.883); however, these two groups each showed significant differences from the IIV4 alone group (*p* < 0.001 for BNT162b2 + IIV4 group vs. IIV4 alone group and *p* < 0.001 for BNT162b2 alone group vs. IIV4 alone group). The incidence decreased to <20% in all groups from the fourth day after injection (Figure 2C).

Adverse reactions and severity in each group according to date are shown in Supplementary Figures S1–S3. In all 3 groups, the adverse reaction rate and severity were high on the day of vaccination and on the first day after vaccination; however, the symptoms improved after the fourth day. In the BNT162b2 + IIV4 group, there were participants who experienced the highest severity of chills, fatigue, myalgia, arthralgia, and abdominal pain on the seventh day after vaccination. In the BNT162b2 alone group, there were participants who developed the highest severity of adverse reactions such as arthralgia, abdominal pain, and injection site pain lasting up to 7 days after vaccination. In the IIV4 alone group, there were no cases of the highest severity of adverse reactions lasting up to 7 days.

### 3.4. Adverse Reactions According to Sex and Age for Each Type of Vaccination

For each type of vaccination, the proportion of adverse reactions according to sex did not significantly differ (Supplementary Tables S1–S3).

When BNT162b2 and IIV4 were vaccinated together, fever occurred in 33.5% of HCWs in their 20s, 15.5% of those in their 30s, 9.1% of those in their 40s, and 6.3% of those in their 50s. The younger the age, the more frequently fever occurred as an adverse reaction, which showed a statistically significant difference. The incidence of other adverse reactions did not statistically differ according to age group (Supplementary Table S4).

In the BNT162b2 alone group, fever more frequently occurred in the younger age groups (50.0% of participants in their 20s, 31.6% of those in their 30s, 13.3% of those in their 40s, and 12.5% of those in their 50s), albeit without statistical significance (*p* = 0.059). The proportion of other adverse reactions according to age group did not show a significant difference (Supplementary Table S5). Additionally, there was no difference in the proportion of adverse reactions according to age group in the IIV4 alone group (Supplementary Table S6).

### 3.5. Adverse Reactions According to Experience with Adverse Reactions after a Previous COVID-19 Vaccination

In participants who experienced fever following a previous COVID-19 vaccination, the relative risk of developing fever after vaccination was 6.97 (95% confidence interval [CI], 2.92–16.62) in the BNT162b2 + IIV4 group and 21.00 (95% CI, 4.21–104.84) in the BNT162b2 alone group. There was no statistical significance among vaccinated participants in the IIV4 alone group (Table 3). With regard to local reactions after vaccination, no significant correlation was observed with whether or not local reactions occurred after a previous COVID-19 vaccination (Table 4).

### 3.6. Medically Attended Adverse Events after BNT162b2 Booster Vaccination

Among 1460 HCWs who consented to this study, 10 cases of medically attended adverse events (4 males and 6 females; 3 patients in their 20s, 5 patients in their 30s, and 2 patients in their 50s) were identified (Table 5). The chief complaints at hospital visits were fever (n = 4), chest pain (n = 2), bruises in both lower extremities (n = 1), right axillar pain (n = 1), syncope (n = 1), and lower-limb weakness (n = 1). The majority of these symptoms appeared between 0 and 5 days after vaccination, and most patients completely recovered after symptomatic treatment.

Notably, a 52-year-old female patient was kept under careful observation. She received the BNT162b2 booster dose at 7 days after influenza vaccination. The influenza vaccination passed without any adverse reaction; however, symptoms such as fever, dizziness, and muscle pain emerged at 2 days after the BNT162b2 booster vaccination. Despite symptomatic treatment, the symptoms persisted. On the 16th day after vaccination, she was hospitalized because of lower-limb weakness and voiding difficulties. She was suspected of having vaccine-induced myelitis, underwent a workup, and was diagnosed with neuromyelitis optica (MNO). She received corticosteroid pulse therapy, plasmapheresis, and rituximab therapy. She has almost recovered and is undergoing rehabilitation.

## 4. Discussion

The importance of the third dose of COVID-19 vaccination continues to be underscored while the COVID-19 epidemic persists worldwide [10]. In Korea, the third dose of vaccination was initiated in October 2021. Safety is particularly important to maximize COVID-19 prevention via an increase in the vaccination rate; nonetheless, little is known about this matter. In particular, as far as we know, no published studies have reported real-world data on adverse reactions that occur when a seasonal influenza vaccine is administered together with a booster vaccine in Korea. Therefore, the present study investigated the adverse reactions occurring after BNT162b2 booster vaccination, particularly when performed together with influenza vaccination. The overall adverse reaction rates were 90.5%, 90.4%, and 44.1% in the BNT162b2 + IIV4 group, BNT162b2 alone group, and IIV4 alone group, respectively.

As shown in this study, the incidence of adverse reactions was higher when the two vaccines were administered together than when the influenza vaccine was administered alone. A previous study on adverse reactions among Korean tertiary hospital workers after influenza vaccination in 2008 reported incidence rates of 33.4%, 17.7%, and 17.0% for injection site pain, myalgia, and fatigue, respectively [11]. Contrastingly, the safety results of BNT162b2 phase 1, 2, and 3 clinical trials revealed incidence rates of 78% and 74% for injection site pain after the first and second doses, respectively, and 43% and 58% for fatigue after the first and second doses, respectively [12]. This difference is thought to be due to a difference in reactogenicity, which is a subset of reactions that occur after vaccination [13]. Similarly, in the present study, BNT162b2 vaccination had a higher incidence of adverse reactions than influenza vaccination. Notably, the frequency of adverse reactions occurring after BNT162b2 vaccination within 1 week of influenza vaccination and after BNT162b2 vaccination alone should be given attention. The results of this study indicated that the difference in the frequency of adverse events was not significant between the BNT162b2 + IIV4 group and BNT162b2 alone group; in particular, the frequency of most severe (grade 4) adverse events was not higher in the BTN162b2 + IIV4 group than in the BNT162b2 alone group. After the third dose, two of participants in this study suffered from abdominal pain and diarrhea with grade 4 severity, which persisted up to 7 days after vaccination. However, this study was conducted on participants using a self-reported questionnaire and the results of the follow-up medical survey indicated no serious problem requiring hospitalization, thus these results could be attributable to individual differences in the perception of discomfort. In this regard, it could be deemed that combined influenza and BNT162b2 vaccinations did not significantly differ from mRNA single vaccination with respect to safety.

As reported in Israel, adverse reactions occurring after the third dose were not more acute than those after the first and second doses, and the safety profile did not appear to significantly differ from the first and second doses [14]. However, it should be noted that the frequency of adverse reactions after the third dose in this study was higher than the frequency of adverse reactions after the second dose reported by phase 1, 2, and 3 clinical trials of BNT162b2 vaccination (local reactions in 4–78% and systemic reactions in 1–58%) [12]. The results of previous studies suggested that the frequency and severity of systemic adverse reactions after the second dose tended to increase compared to those after the first dose. [12,15] In this regard, a more robust immune response might have been induced upon subsequent administration, whereas immunity to the antigen might have been established after the first administration. [16,17] In light of the results of the present study, it can be considered that immunity to the antigen generated by the first and second doses can generate a greater response after the third dose.

Adverse reactions after the first and second doses have been less reported in the elderly and men than in young adults and women. [8,18] The difference in the frequency of adverse reactions according to sex can be attributed to differences in biological and behavioral factors [19] or differences in the perceived discomfort level according to sex [17]. However, the results of this study indicated no significant differences according to age and sex. It can be considered that these results are different from those of previous studies because the proportion of people who knew how to control their discomfort was higher in the current study than in previous studies. In this study, after the third dose of BNT162b2, fever tended to occur more frequently in the younger age groups; this may be due to differences in the immune response with age. However, additional research on this matter is required.

In this study, the risk of developing a fever after the third dose was higher in participants who experienced a fever after a previous COVID-19 vaccination, which may be attributable to differences in the degree of individual immune response to foreign antigens [20]. The results of this study are expected to serve as basic data that can aid in predicting and responding to adverse reactions during medical consultation prior to booster vaccination.

Among the participants of this study, 10 out of 1460 vaccinated HCWs visited a doctor after BNT162b2 booster vaccination. Five of them visited a doctor because of fever and myalgia after injection, although their symptoms improved after symptomatic treatment. Two of them visited the emergency room for chest pain, but there was no evidence of myocarditis or pericarditis after vaccination. These symptoms also improved with symptomatic treatment. Although myocarditis and pericarditis are rare potential complications of COVID-19 mRNA vaccines, including BNT162b2, the Advisory Committee on Immunization Practices for the Centers for Disease Control (CDC) in the United States has recommended continued use, having concluded that the benefits outweigh the risks [21]. A study that analyzed cases of adverse events of vaccines registered in the CDC COVID Data Tracker and Vaccines Adverse Event Reporting System from November 2020 to March 2022 reported that receiving a booster dose of the COVID-19 mRNA vaccine did not increase the risk of myocarditis/pericarditis compared to the primary series [22].

However, one participant was treated for symptoms such as dizziness, febrile sense, and headache after injection, which did not improve. Additionally, lower-limb weakness appeared approximately 2 weeks after receiving the booster dose. She is currently undergoing treatment for NMO from a neurologist. Acute myelitis is a focal inflammatory disorder of the spinal cord but is rarely reported to occur after vaccination. A multi-analysis study conducted by Agmon-Levin et al. in 2009 reported 43 cases of acute myelitis from 1971 to 2007. The types of vaccinations related to myelitis were diverse, including hepatitis B, influenza, polio, and rabies [23]. According to American Neurological Association Investigates, cases of myelitis following COVID-19 vaccination are very rare [24] and several cases have been reported for each vaccine type [25,26,27]. Generally, myelitis occurring after vaccination is believed to be caused by mechanisms such as epitope spreading, cytokine upregulation, and polyclonal activation of lymphocytes by antigens and adjuvants [28]. Nonetheless, because the mRNA-based COVID-19 vaccine does not have an adjuvant and the mRNA is wrapped around lipid nanoparticles and transported, the mechanism underlying the abovementioned neurological pathophysiology remains unclear. Cross-reactivity between the SARS-CoV-2 spike protein antibody and tissue protein may be responsible for demyelination [29] or interaction may occur between the spike protein and ACE2 receptor in endothelial cells in spinal neurons [30]; however, continuous research is required. Considering that the patient in this study showed symptoms of dizziness immediately after vaccination and complained of continuous pain, which occurred after receiving the BNT162b2 booster dose, the temporal relationship between vaccination and myelitis can be considered to be reasonable. Additionally, there is evidence that antigens in vaccines can induce exaggerated autoimmune reactions, thereby causing demyelination of the central nervous system (CNS) [31], and cases of acute demyelinating diseases of CNS such as NMO have recently been reported. Therefore, in the case of this patient, the possibility that myelitis, the first clinical manifestation, is the beginning of NMO due to vaccination can be fully considered [32,33,34]. Nevertheless, such causality still requires confirmation, and further research regarding the underlying mechanism is needed.

The present study had some limitations. First, as this is a study focused on HCWs with relatively high medical knowledge, the adverse reaction rate reported in this study might differ from that in the general public. However, given that the adverse reactions and sensitivity to discomfort reported by HCWs were more accurate, the reported results of this study investigating adverse reactions are considered to be of sufficient significance. Second, because adverse reactions were retrospectively investigated using a mobile questionnaire provided 7 days after each vaccination, bias due to memory distortion could not be excluded. For instance, symptomatic relief after 7 days might underestimate the frequency and severity of adverse events. To minimize this error, the authors thoroughly explained the process of obtaining consent for the study and provided guidance for sincere answers. In addition, the reliability of reported fevers may be called into question since they were assessed through self-reported surveys. However, the study participants, being healthcare workers in a tertiary hospital, had a high understanding of fever and the researchers provided clear instructions for accurate reporting using highly reliable temperature measuring instruments, so the data used in the study can be considered sufficiently reliable. Finally, this study was conducted at a single institution. This study investigated adverse reactions by analyzing medical records for 7 days after vaccination, which was not sufficient to judge the safety of the third dose of BNT162b2.

Nevertheless, this study is meaningful because it is the first study to investigate adverse reactions after BNT162b2 booster vaccination with IIV4 in those vaccinated for the first time as the third dose was implemented in Korea. In particular, the fact that it was possible to indirectly investigate the safety of BNT162b2 booster vaccination with influenza vaccination can be considered a great strength of this study.

## 5. Conclusions

In conclusion, adverse reactions occurred at a significantly higher frequency after the third dose of BNT162b2 vaccine than after the IIV4 vaccine. Additionally, the frequency of adverse reactions one week after vaccination with the third dose of BNT162b2 and IIV4 vaccine was not different from that after vaccination with BNT162b2 alone. The results of this study are expected to serve as basic data regarding the safety of COVID-19 vaccination when performed with influenza vaccination at a time when the SARS-CoV-2 endemic is expected. Continuous and prospective monitoring in the general population is required.

## Figures and Tables

**Table 1:** Baseline characteristics and adverse reactions according to the type of vaccination.

Characteristics	Total (*n* = 394)	BNT162b2 + IIV4 (*n* = 159)	BNT162b2 Alone (*n* = 52)	IIV4 Alone (*n* = 184)	*p* Value
Age	34.85 ± 8.69	35.68 ± 9.21	39.27 ± 9.68	34.12 ± 9.32	<0.001
Sex					
Male	88 (22.3)	30 (18.9)	17 (32.7)	41 (22.3)	0.115
Female	306 (77.7)	129 (81.1)	35 (67.3)	143 (77.7)	
Comorbidities					
Diabetes	5 (1.3)	3 (1.9)	1 (1.9)	1 (0.5)	0.351
Hypertension	19 (4.8)	11 (6.9)	2 (3.8)	6 (3.3)	0.291
Cardiac disease	1 (0.3)	1 (0.6)	0 (0.0)	0 (0.0)	0.534
Neurovascular disease	0 (0.0)	0 (0.0)	0 (0.0)	0 (0.0)	NA
Cancer	7 (1.8)	5 (3.1)	1 (1.9)	1 (0.5)	0.144
Autoimmune disease	7 (1.8)	2 (1.3)	0 (0.0)	5 (2.7)	0.489
Chronic respiratory disease	1 (0.3)	1 (0.6)	0 (0.0)	0 (0.0)	0.534
Morbid obesity	6 (1.5)	3 (1.9)	0 (0.0)	3 (1.6)	0.999
Others	18 (4.6)	7 (4.4)	3 (5.8)	8 (4.3)	0.890
Previous adverse reactions					
Fever	92 (23.4)	33 (20.8)	12 (23.1)	47 (25.5)	0.578
Systemic reactions ^a^	292 (74.1)	121 (76.1)	41 (78.8)	130 (70.7)	0.356
Local reactions ^b^	152 (38.6)	62 (39.0)	27 (51.9)	63 (34.2)	0.068

All values are presented as mean ± standard deviation or as *n* (%). *p* values were calculated using Chi-square or Fisher’s exact tests. ^a^ Headache, chills, fatigue, myalgia, arthralgia, dizziness, nausea and vomiting, chest discomfort, abdominal discomfort, diarrhea, and anxiety/depression. ^b^ Pain, redness, and swelling at the injection site. IIV4 = inactivated influenza vaccine, quadrivalent, NA = not applicable.

**Table 2:** Adverse reactions according to the type of vaccination.

Adverse Reactions	Total (*n* = 394)	BNT162b2 + IIV4 (*n* = 159)	BNT162b2 Alone (*n* = 52)	IIV4 Alone (*n* = 184)	*p* Value
Total	272 (69.0)	144 (90.6) ^a^	47 (90.4) ^a^	81 (44.1) ^b^	<0.001
Fever	51 (12.9)	31 (19.5) ^a^	14 (26.9) ^a^	6 (3.3) ^b^	<0.001
Total systemic reactions	237 (60.2)	124 (78.0) ^a^	42 (80.8) ^a^	71 (38.6) ^b^	<0.001
Headache	148 (37.6)	84 (52.8) ^a^	25 (48.1) ^a^	39 (21.2) ^b^	<0.001
Chills	115 (29.2)	72 (45.3) ^a^	24 (46.2) ^a^	19 (10.3) ^b^	<0.001
Fatigue	231 (58.6)	119 (74.8) ^a^	41 (78.8) ^a^	71 (38.6) ^b^	<0.001
Myalgia	226 (57.4)	123 (77.4) ^a^	42 (80.8) ^a^	61 (33.2) ^b^	<0.001
Arthralgia	69 (17.5)	43 (27.0) ^a^	20 (38.5) ^a^	6 (3.3) ^b^	<0.001
Dizziness	68 (17.3)	41 (25.8) ^a^	16 (30.8) ^a^	11 (6.0) ^b^	<0.001
Nausea	46 (11.7)	28 (17.6) ^a^	12 (23.1) ^a^	6 (3.3) ^b^	<0.001
Vomiting	12 (3.0)	8 (5.0)	0 (0.0)	4 (2.2)	0.132
Chest discomfort	25 (6.3)	15 (9.4) ^a^	5 (9.6) ^a^	5 (2.7) ^b^	0.041
Abdominal discomfort	12 (3.0)	7 (4.4)	3 (5.8)	2 (1.1)	0.059
Diarrhea	22 (5.6)	11 (6.9)	4 (7.7)	7 (3.8)	0.276
Anxiety/depression	17 (4.3)	7 (4.4) ^a^	6 (11.5) ^b^	4 (2.2) ^c^	0.017
Total local reactions	262 (66.5)	144 (90.6) ^a^	47 (90.4) ^a^	71 (38.6) ^b^	<0.001
Pain	256 (65.0)	141 (88.7) ^a^	46 (88.5) ^a^	69 (37.5) ^b^	<0.001
Redness	67 (17.0)	42 (26.4) ^a^	12 (23.1) ^a^	13 (7.1) ^b^	<0.001
Swelling	83 (21.1)	47 (29.6) ^a^	13 (25.0) ^a^	23 (12.5) ^b^	<0.001

All values are presented as *n* (%). *p* values were calculated using the chi-square test, followed by a post-hoc analysis. Percentages within a column followed by the same letter are not significantly different (*p* ≥ 0.05). IIV4 = inactivated influenza vaccine, quadrivalent.

**Table 3:** Frequency of fever and odds ratio according to fever after a previous vaccination.

	Total	Fever	No Fever	Odds Ratio	95% CI	*p* Value
BNT162b2 + IIV4						
Fever after a previous vaccination	33 (100)	16 (48.5)	17 (51.5)	6.97	2.92–16.62	<0.001
No fever after a previous vaccination	126 (100)	15 (11.9)	111 (88.1)	1 (ref)		
BNT162b2 alone						
Fever after a previous vaccination	12 (100)	9 (75.0)	4 (25.0)	21.00	4.21–104.84	<0.001
No fever after a previous vaccination	40 (100)	5 (12.5)	35 (87.5)	1 (ref)		
IIV4 alone						
Fever after a previous vaccination	47 (100)	3 (6.4)	44 (93.6)	3.05	0.59–15.64	0.126
No fever after a previous vaccination	137 (100)	3 (2.2)	134 (97.8)	1 (ref)		

All values are presented as *n* (%), except for the odds ratio and 95% CI. *p* values were calculated by logistic regression analysis. CI = confidence interval, IIV4 = inactivated influenza vaccine, quadrivalent.

**Table 4:** Frequency of local reactions and odds ratios according to local reactions after a previous vaccination.

	Total	Local Reactions	No Local Reactions	Odds Ratio	95% CI	*p* Value
BNT162b2 + IIV4						
Local reactions after a previous vaccination	62 (100)	59 (95.2)	3 (4.8)	2.78	0.75–10.27	0.187
No local reactions after a previous vaccination	97 (100)	85 (87.6)	12 (12.4)	1 (ref)		
BNT162b2 alone						
Local reactions after a previous vaccination	27 (100)	25 (92.6)	2 (7.4)	1.71	0.26–11.16	0.756
No local reactions after a previous vaccination	25 (100)	22 (88.0)	3 (12.0)	1 (ref)		
IIV4 alone						
Local reactions after a previous vaccination	63 (100)	27 (42.9)	36 (57.1)	1.36	0.73–2.54	0.094
No local reactions after a previous vaccination	121 (100)	43 (35.5)	78 (64.5)	1 (ref)		

All values are presented as *n* (%), except for the odds ratio and 95% CI. *p* values were calculated by logistic regression analysis. CI = confidence interval, IIV4 = inactivated influenza vaccine, quadrivalent.

**Table 5:** Characteristics of medically attended adverse events.

Age	Sex	Vaccination Type	Complaint	Symptom Onset after Vaccination, Days	Treatment	Clinical Course
37	Female	BNT162b2 + IIV4	Fever	1	OPD	Recovery
26	Male	BNT162b2 + IIV4	Fever	0	ER	Recovery
31	Female	BNT162b2 + IIV4	Fever	1	ER	Recovery
36	Male	BNT162b2 + IIV4	Fever	1	ER	Recovery
28	Female	BNT162b2 + IIV4	Chest pain	1	ER	Recovery
35	Male	BNT162b2 + IIV4	Chest pain	4	ER	Recovery
27	Female	BNT162b2 + IIV4	Bruises in both legs	5	OPD	Recovery
31	Female	BNT162b2 + IIV4	Right axillar pain	1	OPD	Recovery
55	Male	BNT162b2 + IIV4	Syncope	0	ER	Recovery
52	Female	BNT162b2 + IIV4	Fever Lower-limb weakness	2 16	Hospitalization	Partial recovery ^a^

^a^ Following symptom onset, vaccine-induced myelitis was suspected, and corticosteroid pulse therapy was administered. Subsequently, the occurrence of optic neuritis and neuromyelitis optica-IgG antibody-positive findings were confirmed, and the patient is currently undergoing rehabilitation. IIV4 = inactivated influenza vaccine, quadrivalent, OPD = outpatient department, ER = emergency room.

## Data Availability

Data that are not presented in the article are available upon reasonable request from the corresponding author.

## Supplementary Materials

The following supporting information can be downloaded at: https://www.mdpi.com/article/10.3390/vaccines11020363/s1, Data S1: Questionnaire sheet; Figure S1: Frequency and severity of adverse reactions according to date in the BNT162b2 + inactivated influenza vaccine, quadrivalent group; Figure S2: Frequency and severity of adverse reactions according to date in the BNT162b2 alone group; Figure S3: Frequency and severity of adverse reactions according to date in the inactivated influenza vaccine, quadrivalent alone group; Table S1: Adverse reactions according to sex in the BNT162b2 + inactivated influenza vaccine, quadrivalent group; Table S2: Adverse reactions according to sex in the BNT162b2 alone group; Table S3: Adverse reactions according to sex in the inactivated influenza vaccine, quadrivalent alone group; Table S4: Adverse reactions according to age groups in the BNT162b2 + inactivated influenza vaccine, quadrivalent group; Table S5: Adverse reactions according to age groups in the BNT162b2 alone group; Table S6: Adverse reactions according to age groups in the inactivated influenza vaccine, quadrivalent alone group.
