# Supplementary material for: Adverse Reactions after BNT162b2 Messenger RNA Vaccination for Coronavirus Disease 2019 in Healthcare Workers Compared with Influenza Vaccination
Source: Vaccines (Basel). 2023 Feb 5;11(2):363. doi: 10.3390/vaccines11020363 (PMC9958848; doi:10.3390/vaccines11020363)
Supplement: Supplementary file 1 [file vaccines-11-00363-s001.zip › Supplementary data_Questionnaire sheet.pdf]

Supplementary Data 1. Questionnaire sheet

This is a questionnaire to monitor local and systemic adverse reactions after vaccination against COVID-19. We collect and use personal information for research and monitoring of all employees as follows. All information is anonymized and fully encrypted prior to analysis. The survey collects employee number, gender, date of birth, underlying disease, presence of adverse reactions to previous vaccinations, type of vaccination, and adverse reactions over 7 days.

I agree to the collection and use of personal information.

① Yes ② No

(Question 1) Please give us some information about yourself.

1-1. Please fill in your employee number. ___________.

1-2. Gender ① Male ② Female

1-3. Date of Birth ________/_________/ _________/ (YYYY / MM / DD)

1-4. Underlying disease (possible to overlap)

☐ Diabetes ☐ Hypertension ☐ Cardiac disease ☐ Neurovascular disease ☐ Cancer

☐ Autoimmune disease ☐ Chronic respiratory disease ☐ Morbid obesity (BMI≥30)

☐ Other _________________________

(Question 2) Have you had any adverse reactions after being vaccinated against COVID-19 before?

2-1. ① No ② Yes (‘If yes, please answer 2-2)

2-2. Please select the one that applies (multiple selections possible)

☐ Fever (≥38.0℃)

☐ Headache ☐ Chills ☐ Fatigue ☐ Myalgia ☐ Arthralgia ☐ Dizziness ☐ Nausea

☐ Vomiting ☐ Allergic reactions ☐ Chest discomfort ☐ Abdominal discomfort

☐ Diarrhea ☐ Cellulitis on injection site ☐ Lymphadenitis ☐ Paralysis

☐ Anxiety/Depression ☐ Other _______________

☐ Injection site pain/redness/swelling

(Question 3) What type of vaccine did you receive?

① Influenza vaccination + COVID+19 vaccination

② Influenza vaccination alone

③ COVID-19 vaccination alone

(Question 4) Have you experienced any adverse reactions within 7 days after vaccination?

① No (If no, the survey ends)

②Yes (If yes, please answer the questions below)

(Question 5) Did you have a fever for 7 days from the date of vaccination? If you have experienced it, please select the severity and the date.

5-1. ① No ② 38.0-38.4 °C ③ 38.5-38.9 °C ④ 39.0-39.9 °C ⑤ >40.0°C

5-2. Date (multiple selections possible)

□ Day 0 (The day of vaccination)

□ Day 1 □ Day 2 □ Day 3 □ Day 4 □ Day 5 □ Day 6 □ Day7

(Question 6) Did you take antipyretics for 7 days from the date of vaccination?? If so, please select the type of medicine and the date of taking it.

6-1. Type of antipyretics

① No

② Acetaminophen

③ NSAIDs

④ Both acetaminophen and NSAIDs

6-2. Date of taking antipyretic (multiple selections possible)

□ Day 0 (The day of vaccination)

□ Day 1 □ Day 2 □ Day 3 □ Day 4 □ Day 5 □ Day 6 □ Day7

The following items are a symptom checklist with a severity score. If you have experienced the following symptoms for 7 days after vaccination, please check the severity at the time you consider the most severe. And please check the date (regardless of severity) you had symptoms. Please grade the severity of adverse events as following criteria

- Mild (transient or mild discomfort, no interference with daily activity, and no requirement of medical intervention or therapy)
- Moderate (mild-to-moderate limitation in daily activity, and no or minimal requirement of medical intervention or therapy)
- Severe (substantial limitation in daily activity and requirement of medical intervention or therapy)
- Potentially life-threatening (requires assessment in the emergency department or admission to hospital).
-

(Question 7) Headache

7-1 Have you experienced it?

① No ("If no, go to question 8.)

② Yes (If yes, please answer the questions below)

7-2. Severity

① Mild ② Moderate ③ Severe ④ Potentially life-threatening

7-3. Date of taking antipyretic (multiple selections possible)

☐ Day 0 (The day of vaccination)

☐ Day 1 ☐ Day 2 ☐ Day 3 ☐ Day 4 ☐ Day 5 ☐ Day 6 ☐ Day7

(Question 8) Chills

8-1 Have you experienced it?

① No (“If no, go to question 9.)

② Yes (If yes, please answer the questions below)

8-2. Severity

① Mild ② Moderate ③ Severe ④ Potentially life-threatening

8-3. Date of taking antipyretic (multiple selections possible)

☐ Day 0 (The day of vaccination)

☐ Day 1 ☐ Day 2 ☐ Day 3 ☐ Day 4 ☐ Day 5 ☐ Day 6 ☐ Day7

(Question 9) Fatigue

9-1 Have you experienced it?

① No (“If no, go to question 10)

② Yes (If yes, please answer the questions below)

9-2. Severity

① Mild ② Moderate ③ Severe ④ Potentially life-threatening

9-3. Date of taking antipyretic (multiple selections possible)

☐ Day 0 (The day of vaccination)

☐ Day 1 ☐ Day 2 ☐ Day 3 ☐ Day 4 ☐ Day 5 ☐ Day 6 ☐ Day7

(Question 10) Myalgia

10-1 Have you experienced it?

① No (“If no, go to question 11)

② Yes (If yes, please answer the questions below)

10-2. Severity

① Mild ② Moderate ③ Severe ④ Potentially life-threatening

10-3. Date of taking antipyretic (multiple selections possible)

☐ Day 0 (The day of vaccination)

☐ Day 1 ☐ Day 2 ☐ Day 3 ☐ Day 4 ☐ Day 5 ☐ Day 6 ☐ Day7

(Question 11) Arthralgia

11-1 Have you experienced it?

① No (“If no, go to question 12)

② Yes (If yes, please answer the questions below)

11-2. Severity

① Mild ② Moderate ③ Severe ④ Potentially life-threatening

11-3. Date of taking antipyretic (multiple selections possible)

☐ Day 0 (The day of vaccination)

☐ Day 1 ☐ Day 2 ☐ Day 3 ☐ Day 4 ☐ Day 5 ☐ Day 6 ☐ Day7

(Question 12) Dizziness

12-1 Have you experienced it?

① No (“If no, go to question 13)

② Yes (If yes, please answer the questions below)

12-2. Severity

① Mild ② Moderate ③ Severe ④ Potentially life-threatening

12-3. Date of taking antipyretic (multiple selections possible)

☐ Day 0 (The day of vaccination)

☐ Day 1 ☐ Day 2 ☐ Day 3 ☐ Day 4 ☐ Day 5 ☐ Day 6 ☐ Day7

(Question 13) Nausea

13-1 Have you experienced it?

① No (“If no, go to question 14)

② Yes (If yes, please answer the questions below)

13-2. Severity

① Mild ② Moderate ③ Severe ④ Potentially life-threatening

13-3. Date of taking antipyretic (multiple selections possible)

☐ Day 0 (The day of vaccination)

☐ Day 1 ☐ Day 2 ☐ Day 3 ☐ Day 4 ☐ Day 5 ☐ Day 6 ☐ Day7

(Question 14) Vomiting

14-1 Have you experienced it?

① No (“If no, go to question 15)

② Yes (If yes, please answer the questions below)

14-2. Severity

① Mild ② Moderate ③ Severe ④ Potentially life-threatening

14-3. Date of taking antipyretic (multiple selections possible)

☐ Day 0 (The day of vaccination)

☐ Day 1 ☐ Day 2 ☐ Day 3 ☐ Day 4 ☐ Day 5 ☐ Day 6 ☐ Day7

(Question 15) Chest discomfort

15-1 Have you experienced it?

① No (“If no, go to question 16)

② Yes (If yes, please answer the questions below)

15-2. Severity

① Mild ② Moderate ③ Severe ④ Potentially life-threatening

15-3. Date of taking antipyretic (multiple selections possible)

□ Day 0 (The day of vaccination)

□ Day 1 □ Day 2 □ Day 3 □ Day 4 □ Day 5 □ Day 6 □ Day7

(Question 16) Abdominal discomfort

16-1 Have you experienced it?

① No (“If no, go to question 17)

② Yes (If yes, please answer the questions below)

16-2. Severity

② Mild ② Moderate ③ Severe ④ Potentially life-threatening

16-3. Date of taking antipyretic (multiple selections possible)

□ Day 0 (The day of vaccination)

□ Day 1 □ Day 2 □ Day 3 □ Day 4 □ Day 5 □ Day 6 □ Day7

(Question 17) Diarrhea

17-1 Have you experienced it?

① No (“If no, go to question 18)

② Yes (If yes, please answer the questions below)

17-2. Severity

① Mild ② Moderate ③ Severe ④ Potentially life-threatening

17-3. Date of taking antipyretic (multiple selections possible)

☐ Day 0 (The day of vaccination)

☐ Day 1 ☐ Day 2 ☐ Day 3 ☐ Day 4 ☐ Day 5 ☐ Day 6 ☐ Day7

(Question 18) Anxiety/Depression

18-1 Have you experienced it?

① No (“If no, go to question 19)

① Yes (If yes, please answer the questions below)

18-2. Severity

① Mild ② Moderate ③ Severe ④ Potentially life-threatening

18-3. Date of taking antipyretic (multiple selections possible)

☐ Day 0 (The day of vaccination)

☐ Day 1 ☐ Day 2 ☐ Day 3 ☐ Day 4 ☐ Day 5 ☐ Day 6 ☐ Day7

(Question 19) Pain of injection site

19-1 Have you experienced it?

① No (“If no, go to question 20)

② Yes (If yes, please answer the questions below)

19-2. Severity

① Mild ② Moderate ③ Severe ④ Potentially life-threatening

19-3. Date of taking antipyretic (multiple selections possible)

☐ Day 0 (The day of vaccination)

☐ Day 1 ☐ Day 2 ☐ Day 3 ☐ Day 4 ☐ Day 5 ☐ Day 6 ☐ Day7

(Question 20) Redness of injection site (Except for minor redness less than 2 cm)

10-1 Have you experienced it?

① No (“If no, go to question 21)

② Yes (If yes, please answer the questions below)

20-2. Severity

① 2.0 to 5.0 cm in diameter; larger than the size of a coin

② >5.0 to 10.0 cm in diameter; larger than the size of an egg

③ >10.0 cm in diameter; larger than the size of a fist

④ necrosis or exfoliative dermatitis

20-3. Date of taking antipyretic (multiple selections possible)

□ Day 0 (The day of vaccination)

□ Day 1 □ Day 2 □ Day 3 □ Day 4 □ Day 5 □ Day 6 □ Day7

(Question 21) Swelling of injection site (Except for minor swelling less than 2 cm)

21-1 Have you experienced it?

① No (“If no, go to question 22)

② Yes (If yes, please answer the questions below)

21-2. 중증도

① 2.0 to 5.0 cm in diameter; larger than the size of a coin

② >5.0 to 10.0 cm in diameter; larger than the size of an egg

③ >10.0 cm in diameter; larger than the size of a fist

④ necrosis

21-3. Date of taking antipyretic (multiple selections possible)

□ Day 0 (The day of vaccination)

□ Day 1 □ Day 2 □ Day 3 □ Day 4 □ Day 5 □ Day 6 □ Day7

(Question 22) Other ______________

22-1 Have you experienced it?

① No (“If no, go to question 8.)

② Yes (If yes, please answer the questions below)

22-2. Severity

① Mild ② Moderate ③ Severe ④ Potentially life-threatening

22-3. Date of taking antipyretic (multiple selections possible)

□ Day 0 (The day of vaccination)

□ Day 1 □ Day 2 □ Day 3 □ Day 4 □ Day 5 □ Day 6 □ Day7
