# Supplementary material for: Adverse Reactions after BNT162b2 Messenger RNA Vaccination for Coronavirus Disease 2019 in Healthcare Workers Compared with Influenza Vaccination
Source: Vaccines (Basel). 2023 Feb 5;11(2):363. doi: 10.3390/vaccines11020363 (PMC9958848; doi:10.3390/vaccines11020363)
Supplement: Supplementary file 1 [file vaccines-11-00363-s001.zip › supplementary table.pdf]

Supplementary Table S1. Adverse reactions according to sex in the BNT162b2 vaccination with inactivated quadrivalent influenza vaccine group.

**Variables**	**Total (n=159)**	**Male (n=30)**	**Female (n=129)**	**P-value**
Fever	31 (19.5)	5 (16.7)	26 (20.2)	0.664
Any systemic reactions	124 (78.0)	22 (73.3)	102 (79.1)	0.491
Headache	84 (52.8)	13 (43.4)	71 (55.)	0.462
Chills	72 (45.3)	14 (46.7)	58 (45.0)	0.401
Fatigue	119 (74.8)	21 (70.0)	98 (76.0)	0.063
Myalgia	123 (77.4)	21 (70.0)	102 (79.1)	0.213
Arthralgia	43 (27.0)	8 (26.7)	35 (27.1)	0.081
Dizziness	41 (25.8)	5 (16.7)	36 (27.9)	0.729
Nausea	28 (17.6)	3 (10.0)	25 (19.4)	0.265
Vomiting	8 (5.0)	1 (3.3)	7 (5.4)	0.999
Chest discomfort	15 (9.4)	1 (3.3)	14 (10.9)	0.306
Abdominal discomfort	7 (4.4)	1 (3.3)	6 (4.7)	0.999
Diarrhea	11 (6.9)	1 (3.0)	10 (7.8)	0.692
Anxiety / Depression	7 (4.4)	0 (0.0)	7 (5.4)	0.348
Any local reactions	144 (90.6)	26 (86.7)	118 (91.5)	0.091
Pain	141 (88.7)	26 (86.7)	115 (89.1)	0.213
Redness	42 (26.4)	8 (26.7)	34 (26.4)	0.875
Swelling	47 (29.6)	9 (30.0)	38 (29.5)	0.841

All values are presented as n (%).

P-values were calculated using the chi-square or Fisher's exact test.

Supplementary Table S2. Adverse reactions according to sex in the BNT162b2 vaccination alone group.

**Variables**	**Total (n=52)**	**Male (n=17)**	**Female (n=35)**	**P-value**
Fever	14 (26.9)	4 (23.5)	10 (28.6)	0.123
Any systemic reactions	42 (80.8)	14 (82.4)	28 (80.0)	0.594
Headache	25 (48.1)	8 (47.1)	18 (48.6)	0.307
Chills	24 (46.2)	8 (47.1)	16 (45.7)	0.551
Fatigue	41 (78.8)	14 (82.4)	27 (77.1)	0.596
Myalgia	42 (80.8)	14 (82.4)	28 (80.0)	0.467
Arthralgia	20 (38.5)	6 (35.3)	14 (40.0)	0.248
Dizziness	16 (30.8)	5 (29.4)	11 (31.4)	0.331
Nausea	12 (23.1)	4 (23.5)	8 (22.9)	0.976
Vomiting	0 (0.0)	0 (0.0)	0	NA
Chest discomfort	5 (9.6)	2 (11.8)	3 (8.6)	0.072
Abdominal discomfort	3 (5.8)	1 (5.9)	2 (5.7)	0.942
Diarrhea	4 (7.7)	1 (5.9)	3 (8.6)	0.617
Anxiety / Depression	6 (11.5)	2 (11.8)	4 (11.4)	0.992
Any local reactions	47 (90;4)	15 (88.2)	32 (91.4)	0.457
Pain	46 (88.5)	15 (88.2)	31 (88.6)	0.885
Redness	12 (23.1)	4 (23.5)	8 (22.9)	0.864
Swelling	13 (25.0)	4 (23.5)	9 (25.7)	0.757

NA, not applicable

All values are presented as n (%).

P-values were calculated using the chi-square or Fisher's exact test.

Supplementary Table S3. Adverse reactions according to sex in the inactivated quadrivalent influenza vaccine alone group.

**Variables**	**Total (n=184)**	**Male (n=41)**	**Female (n=143)**	**P-value**
Fever	6 (3.3)	1 (2.4)	5 (3.5)	0.998
Any systemic reactions	71 (38.6)	14 (34.1)	57 (39.9)	0.363
Headache	39 (21.2)	7 (17.1)	32 (22.4)	0.462
Chills	19 (10.3)	4 (9.8)	15 (10.5)	0.401
Fatigue	71 (38.6)	14 (34.1)	57 (39.9)	0.363
Myalgia	61 (33.2)	12 (29.3)	49 (34.3)	0.151
Arthralgia	6 (3.3)	2 (4.9)	4 (2.8)	0.218
Dizziness	11 (6.0)	2 (4.9)	9 (6.3)	0.729
Nausea	6 (3.3)	2 (4.9)	4 (2.8)	0.218
Vomiting	4 (2.2)	2 (4.9)	2 (1.4)	0.135
Chest discomfort	5 (2.7)	1 (2.4)	4 (2.8)	0.999
Abdominal discomfort	2 (1.1)	1 (2.4)	1 (0.7)	0.399
Diarrhea	7 (3.8)	3 (7.3)	4 (2.8)	0.101
Anxiety / Depression	4 (2.2)	1 (2.4)	3 (2.1)	0.999
Any local reactions	71 (38.6)	13 (31.7)	58 (40.6)	0.097
Pain	69 (37.5)	13 (31.7)	56 (39.2)	0.645
Redness	13 (7.1)	2 (4.9)	11 (7.7)	0.128
Swelling	23 (12.5)	4 (9.8)	19 (13.3)	0.225

All values are presented as n (%).

P-values were calculated using the chi-square or Fisher's exact test.

Supplementary Table S4. Adverse reactions according to age group in the BNT162b2 vaccination with inactivated quadrivalent influenza vaccine group.

**Variables**	**Total (n=159)**	**20s (n=54)**	**30s (n=58)**	**40s (n=33)**	**50s (n=16)**	**60s (n=0)**	**P-value**
Fever	31 (19.5)	18 (33.3)	9 (15.5)	3 (9.1)	1 (6.3)	0 (0.0)	0.009
Any systemic reactions	124 (78.0)	43 (79.6)	49 (84.5)	23 (69.7)	9 (56.3)	0 (0.0)	0.448
Headache	84 (52.8)	27 (50.0)	36 (62.1)	17 (51.5)	4 (25.0)	0 (0.0)	0.137
Chills	72 (45.3)	26 (48.1)	29 (50.0)	13 (39.4)	4 (25.0)	0 (0.0)	0.359
Fatigue	119 (74.8)	40 (74.1)	49 (84.5)	21 (63.6)	9 (56.3)	0 (0.0)	0.127
Myalgia	123 (77.4)	43 (79.6)	49 (84.5)	23 (69.7)	8 (50.0)	0 (0.0)	0.186
Arthralgia	43 (27.0)	9 (16.7)	21 (36.2)	10 (30.3)	3 (18.8)	0 (0.0)	0.099
Dizziness	41 (25.8)	18 (33.)	13 (22.4)	7 (21.2)	3 (18.8)	0 (0.0)	0.439
Nausea	28 (17.6)	5 (9.4)	13 (22.4)	8 (24.2)	2 (12.5)	0 (0.0)	0.278
Vomiting	8 (5.0)	1 (1.9)	5 (8.6)	2 (6.1)	0 (0.0)	0 (0.0)	0.449
Chest discomfort	15 (9.4)	6 (11.1)	5 (8.6)	1 (3.0)	3 (18.8)	0 (0.0)	0.284
Abdominal discomfort	7 (4.4)	3 (5.6)	4 (6.9)	0 (0.0)	0 (0.0)	0 (0.0)	0.495
Diarrhea	11 (6.9)	4 (7.4)	4 (6.9)	3 (9.1)	0 (0.0)	0 (0.0)	0.816
Anxiety / Depression	7 (4.4)	3 (5.6)	3 (5.2)	1 (3.0)	0 (0.0)	0 (0.0)	0.999
Any local reactions	144 (90.6)	49 (90.7)	53 (91.4)	31 (93.9)	11 (68.8)	0 (0.0)	0.788
Pain	141 (88.7)	48 (88.9)	52 (89.7)	31 (93.9)	10 (62.5)	0 (0.0)	0.711
Redness	42 (26.4)	14 (25.9)	18 (31.0)	6 (18.2)	4 (25.0)	0 (0.0)	0.859
Swelling	47 (29.6)	16 (29.6)	20 (34.5)	8 (24.2)	3 (18.8)	0 (0.0)	0.625

All values are presented as n (%).

P-values were calculated using the chi-square or Fisher’s exact test.

Supplementary Table S5. Adverse reactions according to age group in the BNT162b2 vaccination group.

Variables	Total (n=52)	20s (n=10)	30s (n=19)	40s (n=15)	50s (n=8)	60s (n=0)	P-value
Fever	14 (26.9)	5 (50.0)	6 (31.6)	2 (13.3)	1 (12.5)	0 (0.0)	0.059
Any systemic reactions	42 (80.8)	9 (90.0)	15 (78.9)	12 (80.0)	6 (75.0)	0 (0.0)	0.803
Headache	25 (48.1)	5 (50.0)	10 (52.6)	6 (40.0)	4 (50.0)	0 (0.0)	0.356
Chills	24 (46.2)	9 (90.0)	9 (47.4)	6 (40.0)	0 (0.0)	0 (0.0)	0.216
Fatigue	41 (78.8)	8 (14.8)	15 (78.9)	12 (80.0)	6 (75.0)	0 (0.0)	0.686
Myalgia	42 (80.8)	8 (16.7)	16 (84.2)	12 (80.0)	6 (75.0)	0 (0.0)	0.803
Arthralgia	20 (38.5)	1 (10.0)	4 (21.1)	9 (60.0)	6 (75.0)	0 (0.0)	0.001
Dizziness	16 (30.8)	4 (40.0)	5 (26.3)	4 (26.7)	3 (37.5)	0 (0.0)	0.357
Nausea	12 (23.1)	3 (30.0)	4 (21.1)	2 (13.3)	3 (37.5)	0 (0.0)	0.128
Vomiting	0 (0.0)	0 (0.0)	0 (0.0)	0 (0.0)	0 (0.0)	0 (0.0)	NA
Chest discomfort	5 (9.6)	1 (10.0)	3 (15.8)	1 (6.7)	0 (0.0)	0 (0.0)	0.607
Abdominal discomfort	3 (5.8)	0 (0.0)	2 (10.5)	0 (0.0)	1 (12.5)	0 (0.0)	0.404
Diarrhea	4 (7.7)	0 (0.0)	3 (15.8)	0 (0.0)	1 (12.5)	0 (0.0)	0.308
Anxiety / Depression	6 (11.5)	2 (20.0)	4 (21.1)	0 (0.0)	0 (0.0)	0 (0.0)	0.146
Any local reactions	47 (90;4)	9 (90.0)	17 (89.5)	14 (93.3)	7 (87.5)	0 (0.0)	0.818
Pain	46 (88.5)	9 (90.0)	17 (89.5)	14 (93.3)	6 (75.0)	0 (0.0)	0.756
Redness	12 (23.1)	2 (20.0)	5 (26.3)	4 (26.7))	1 (12.5)	0 (0.0)	0.567
Swelling	13 (25.0)	2 (20.0)	5 (26.3)	5 (33.3)	1 (12.5)	0 (0.0)	0.448

NA, not applicable

All values are presented as n (%).

P-values were calculated using the chi-square or Fisher's exact test.

Supplementary Table S6. Adverse reactions according to age group in the inactivated quadrivalent influenza vaccine alone group.

Variables	Total (n=184)	20s (n=74)	30s (n=60)	40s (n=35)	50s (n=13)	60s (n=2)	P-value
Fever	6 (3.3)	5 (6.8)	1 (1.7)	0 (0.0)	0 (0.0)	0 (0.0)	0.322
Any systemic reactions	71 (38.6)	29 (39.2)	25 (41.7)	12 (34.3)	5 (38.5)	0 (0.0)	0.365
Headache	39 (21.2)	17 (23.0)	12 (20.0)	7 (20.0)	3 (23.1)	0 (0.0)	0.228
Chills	19 (10.3)	8 (10.8)	6 (10.0)	4 (11.4)	1 (7.7)	0 (0.0)	0.623
Fatigue	71 (38.6)	29 (39.2)	25 (41.7)	12 (34.3)	5 (38.5)	0 (0.0)	0.365
Myalgia	61 (33.2)	25 (33.8)	23 (38.3)	10 (28.6)	3 (23.1)	0 (0.0)	0.765
Arthralgia	6 (3.3)	2 (2.7)	2 (3.3)	1 (2.9)	1 (7.7)	0 (0.0)	0.624
Dizziness	11 (6.0)	6 (8.1)	3 (5.0)	2 (5.7)	0 (0.0)	0 (0.0)	0.653
Nausea	6 (3.3)	3 (4.1)	2 (3.3)	1 (2.9)	0 (0.0)	0 (0.0)	0.933
Vomiting	4 (2.2)	1 (1.4)	2 (3.3)	1 (2.9)	0 (0.0)	0 (0.0)	0.768
Chest discomfort	5 (2.7)	2 (2.7	2 (3.3)	1 (2.9)	0 (0.0)	0 (0.0)	0.999
Abdominal discomfort	2 (1.1)	1 (1.4)	1 (1.7)	0 (0.0)	0 (0.0)	0 (0.0)	0.999
Diarrhea	7 (3.8)	4 (5.4)	3 (5.0)	0 (0.0)	0 (0.0)	0 (0.0)	0.666
Anxiety / Depression	4 (2.2)	1 (1.4)	1 (1.7)	1 (2.9)	1 (7.7)	0 (0.0)	0.361
Any local reactions	71 (38.6)	29 (39.2)	23 (38.3)	15 (42.9)	4 (30.8)	0 (0.0)	0.774
Pain	69 (37.5)	29 (39.2)	23 (38.3)	13 (37.1)	4 (30.8)	0 (0.0)	0.655
Redness	13 (7.1)	5 (6.8)	5 (8.3)	2 (5.7)	1 (7.7)	0 (0.0)	0.895
Swelling	23 (12.5)	9 (12.2)	8 (13.3)	4 (11.4)	2 (15.4)	0 (0.0)	0.458

All values are presented as n (%).

P-values were calculated using the chi-square or Fisher’s exact test.
