# Supplementary figures and images for: Adverse Reactions after BNT162b2 Messenger RNA Vaccination for Coronavirus Disease 2019 in Healthcare Workers Compared with Influenza Vaccination
Source: Vaccines (Basel). 2023 Feb 5;11(2):363. doi: 10.3390/vaccines11020363 (PMC9958848; doi:10.3390/vaccines11020363)

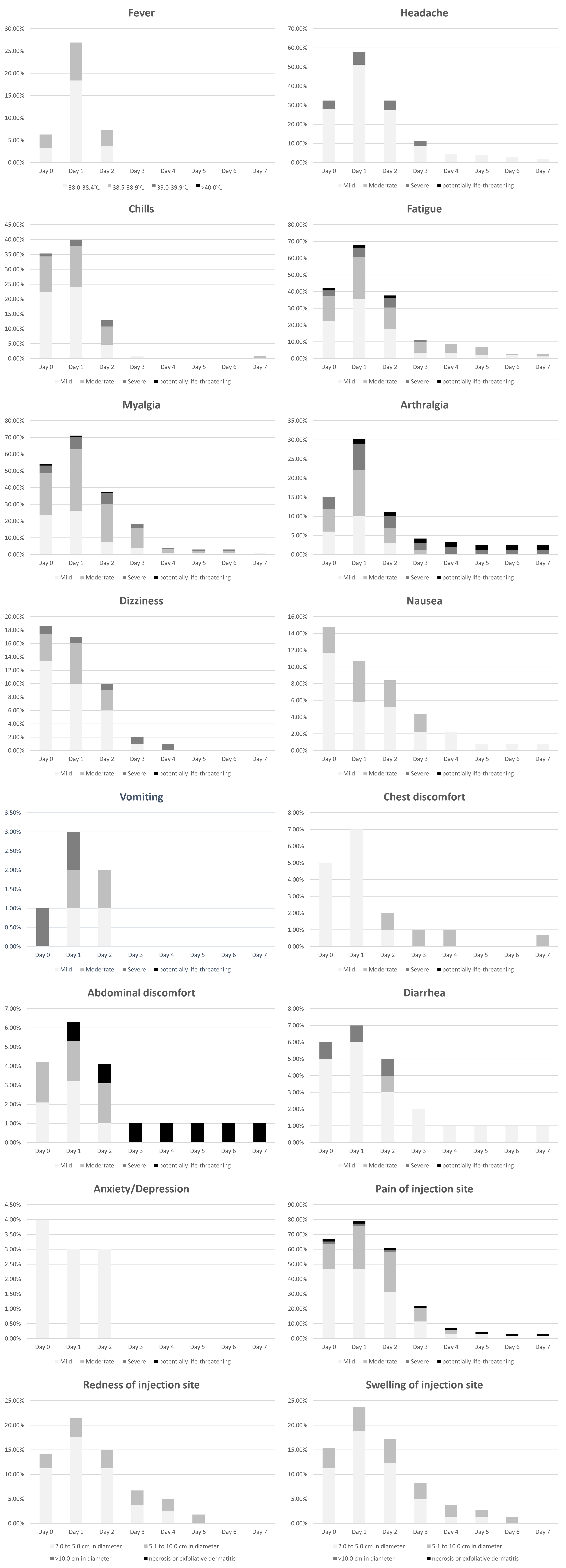

Supplement: Supplementary file 1 [file vaccines-11-00363-s001.zip › Supplementary Figure S2.TIF]

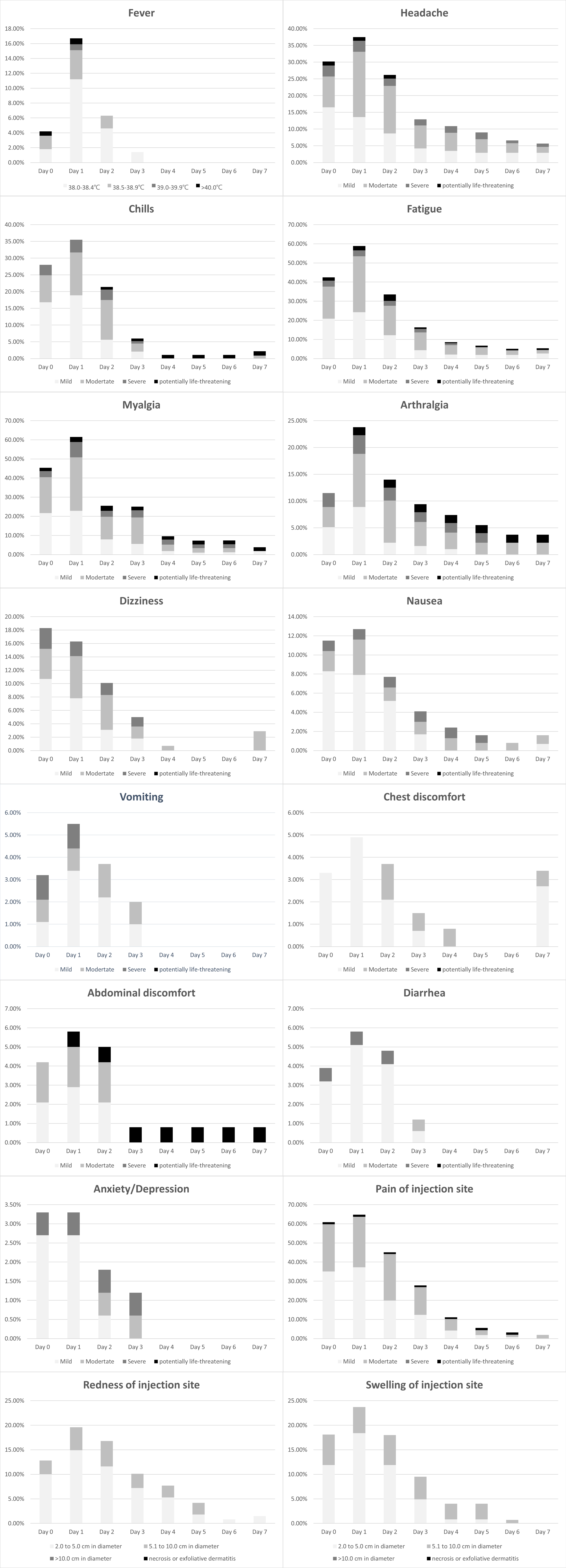

Supplement: Supplementary file 1 [file vaccines-11-00363-s001.zip › Supplemetary Figure S1.TIF]

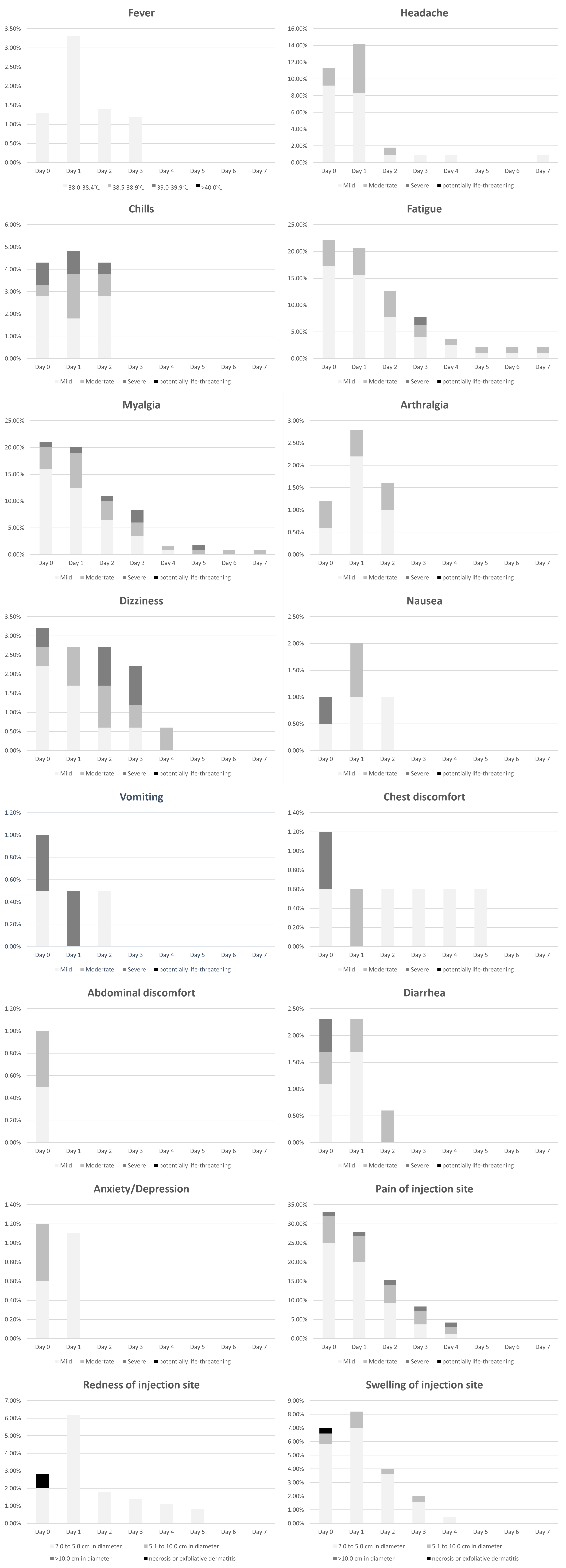

Supplement: Supplementary file 1 [file vaccines-11-00363-s001.zip › Supplemetary Figure S3.TIF]
